# Management of a case of buried bumper syndrome using an endoscopic submucosal dissection-based approach

**DOI:** 10.1055/a-2578-2649

**Published:** 2025-04-17

**Authors:** Ernesto Fasulo, Francesco Vito Mandarino, Alberto Barchi, Giuseppe DellʼAnna, Silvio Danese, Francesco Azzolini

**Affiliations:** 1 Gastroenterology and Gastrointestinal Endoscopy, IRCCS Ospedale San Raffaele and Vita-Salute San Raffaele University, Milan, Italy; 2 27288 Gastroenterology and Gastrointestinal Endoscopy, IRCCS Policlinico San Donato, San Donato Milanese, Italy

Buried bumper syndrome (BBS) is a serious complication of percutaneous endoscopic gastrostomy (PEG) characterized by the internal bumper migrating to the gastric or abdominal wall. Its incidence ranges from 0.3 to 2.4% per PEG-patient per year 1 . Over the years, various strategies for managing BBS have been reported 2 3 4 , including the development of dedicated endoscopic devices (Flamingo Set; Medwork).

We present a case of BBS treated with an endoscopic submucosal dissection (ESD)-based approach ( Video 1 ).

BBS treatment using an ESD-based approach. BBS, buried bumper syndrome; ESD, endoscopic submucosal dissection.Video 1

A 69-year-old male with Parkinson’s disease underwent PEG-jejunal (PEG-J) placement for dopaminergic therapy infusion. Two years later, the PEG-J became non-functional, and the patient was referred to our center. Esophagogastroduodenoscopy revealed a gastric bulge suggestive of BBS ( Fig. 1 ), which was confirmed by a CT scan. Endoscopic removal was planned using a knife-assisted ESD-based approach.

**Fig. 1:**
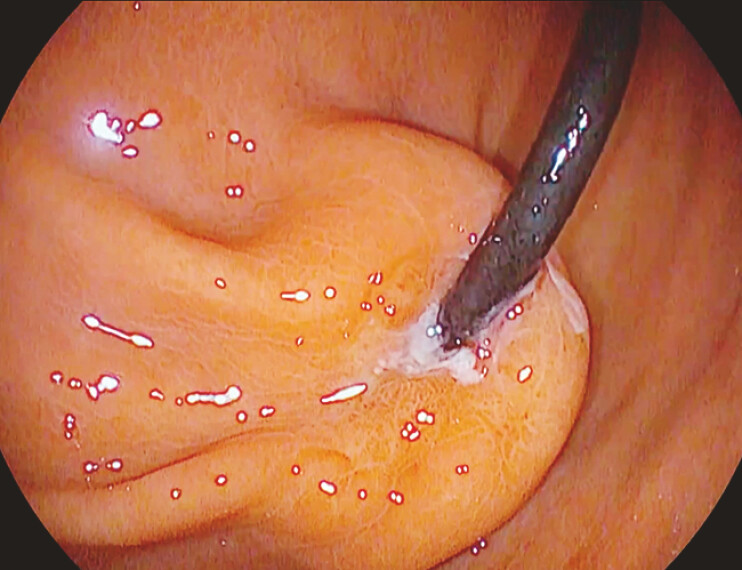
The preliminary endoscopic view consistent with BBS. BBS, buried bumper syndrome.

An initial incision was made near the PEG tube using an L-knife (Finemedix, South Korea) to access the buried bumper bulge. The incision was then progressively widened towards the center to enable mobilization of the tube. Next, an O-knife (Finemedix, South Korea) was used to dissect the surrounding fibrotic tissue ( Fig. 2 ). Once freed, the tube was removed to facilitate further dissection. The residual tissue was excised with a hot snare to improve the visualization and clear the working field ( Fig. 3 ).

**Fig. 2:**
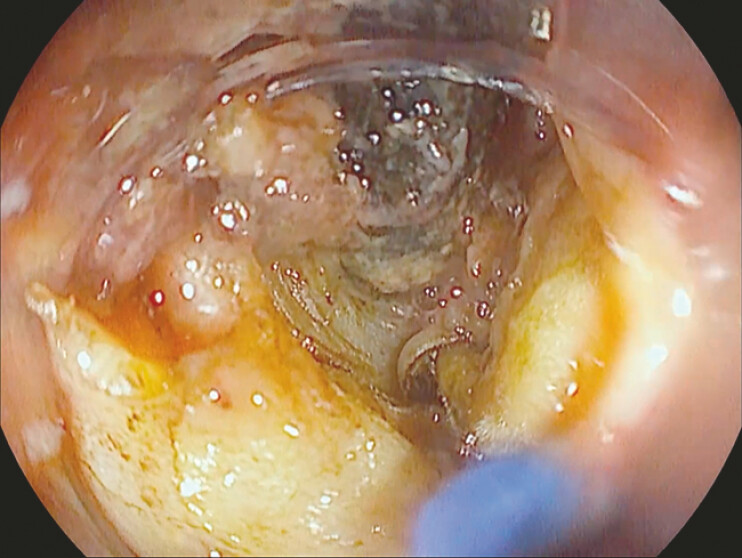
Dissection of the tissue around the PEG tube. PEG, percutaneous endoscopic gastrostomy.

**Fig. 3:**
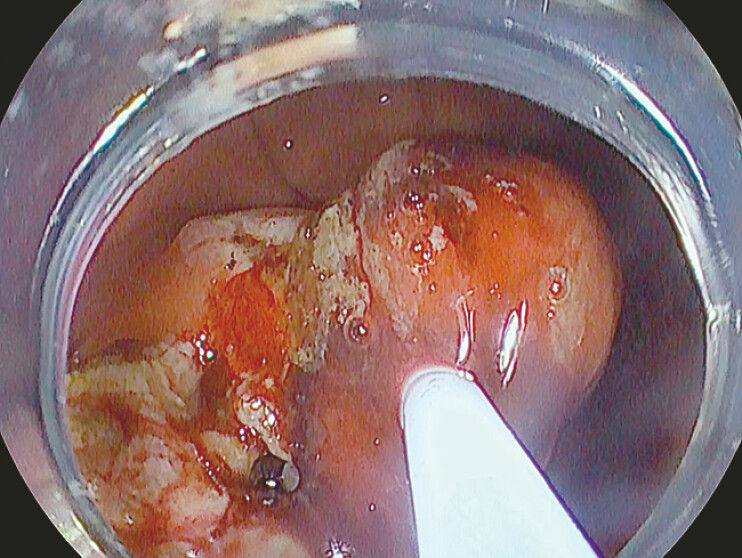
The hot snare used to clear the working field.

Fluoroscopy was utilized during the procedure to guide the dissection and confirm the precise localization of the bumper. Once fully exposed, the bumper was securely grasped with foreign body forceps and extracted transorally ( Fig. 4 ). In the final fluoroscopic assessment, no contrast leakages were observed ( Fig. 5 ).

**Fig. 4:**
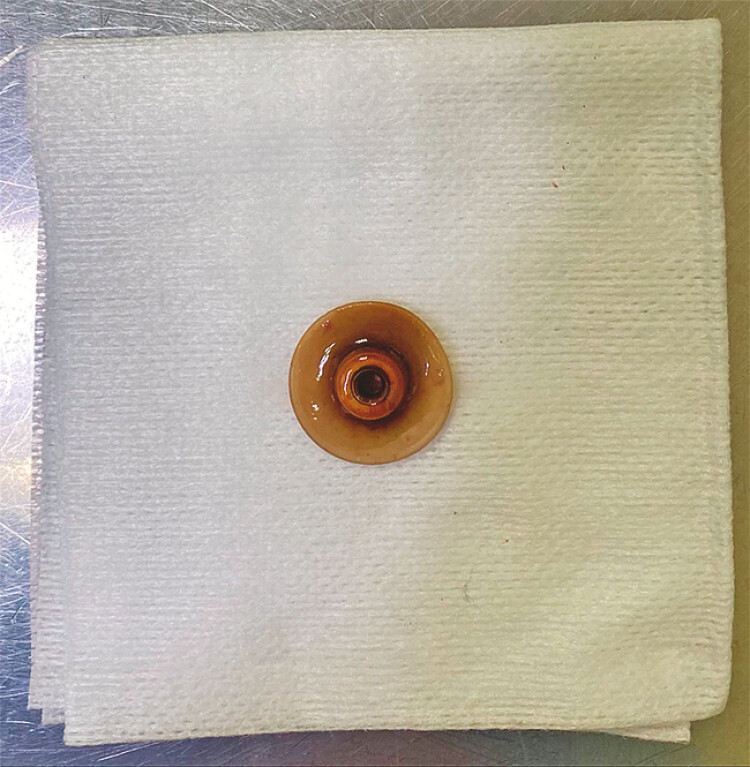
The extracted bumper.

**Fig. 5:**
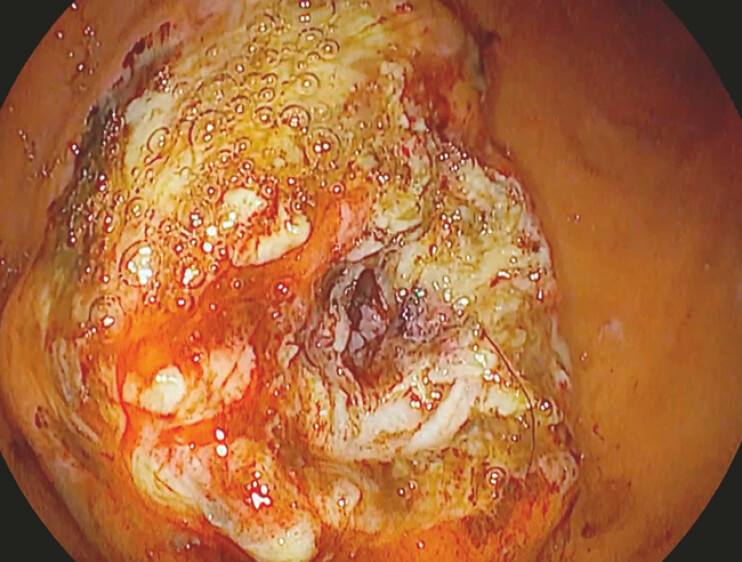
The final endoscopic view of the dissection defect.

The patient was discharged the following day without any complications. Dopaminergic therapy was transitioned to oral formulation.

This case highlights that the knife-assisted ESD technique is a minimally invasive and precise approach, offering a safe and effective solution for the endoscopic management of BBS.

Endoscopy_UCTN_Code_TTT_1AO_2AK

**Correction**

**Correction: Management of a case of buried bumper syndrome using an endoscopic submucosal dissection-based approach** Fasulo Ernesto, Mandarino Francesco Vito, Barchi Alberto et al. Management of a case of buried bumper syndrome using an endoscopic submucosal dissection-based approach. Endoscopy 2025; 57: E321–E322, doi:10.1055/a-2578-2649 In the above-mentioned article the title has been corrected. Correct is the following title: Management of a case of buried bumper syndrome using an endoscopic submucosal dissection-based approach. This was corrected in the online version on May 05, 2025.

## Notes

Endoscopy E-Videos https://eref.thieme.de/e-videos
*E-Videos* is an open access online section of the journal *Endoscopy* , reporting on interesting cases and new techniques in gastroenterological endoscopy. All papers include a high-quality video and are published with a Creative Commons CC-BY license. Endoscopy E-Videos qualify for HINARI discounts and waivers and eligibility is automatically checked during the submission process. We grant 100% waivers to articles whose corresponding authors are based in Group A countries and 50% waivers to those who are based in Group B countries as classified by Research4Life (see: https://www.research4life.org/access/eligibility/ ). This section has its own submission website at https://mc.manuscriptcentral.com/e-videos .

## Notes

Correction **Correction: Management of a case of buried bumper syndrome using an endoscopic submucosal dissection-based approach** Fasulo Ernesto, Mandarino Francesco Vito, Barchi Alberto et al. Management of a case of buried bumper syndrome using an endoscopic submucosal dissection-based approach. Endoscopy 2025; 57: E321–E322, doi:10.1055/a-2578-2649 In the above-mentioned article the title has been corrected. Correct is the following title: Management of a case of buried bumper syndrome using an endoscopic submucosal dissection-based approach. This was corrected in the online version on May 05, 2025.
