# Correction: Management of a case of buried bumper syndrome using an endoscopic submucosal dissection-based approach

**DOI:** 10.1055/a-2596-6400

**Published:** 2025-05-06

**Authors:** Ernesto Fasulo, Francesco Vito Mandarino, Alberto Barchi, Giuseppe DellʼAnna, Silvio Danese, Francesco Azzolini

**Affiliations:** 1 Gastroenterology and Gastrointestinal Endoscopy, IRCCS Ospedale San Raffaele and Vita-Salute San Raffaele University, Milan, Italy; 2 27288 Gastroenterology and Gastrointestinal Endoscopy, IRCCS Policlinico San Donato, San Donato Milanese, Italy



In the above-mentioned article the title has been corrected. Correct is the following title: Management of a case of buried bumper syndrome using an endoscopic submucosal dissection-based approach. This was corrected in the online version on May 05, 2025.

